# Monitoring the Conformance of Patients Undergoing Electroconvulsive Therapy (ECT) Treatment to Electroconvulsive Therapy Accreditation Services (ECTAS) Standards at Worcestershire Specialist Mental Health Services

**DOI:** 10.1192/bjo.2024.649

**Published:** 2024-08-01

**Authors:** Nancy Youssef

**Affiliations:** Herefordshire and Worcestershire Health and Care NHS Trust, Worcester, United Kingdom

## Abstract

**Aims:**

To confirm that 100% of patients treated by Electroconvulsive Therapy (ECT) have weekly assessment of mental state via Montgomery–Åsberg Depression Scale (MADRS).

To confirm that 100% of patients treated with Electroconvulsive Therapy (ECT) have regular assessment of their cognition before treatment and every 4 treatment sessions via the Montreal Cognitive Assessment Scale (MOCA).

**Methods:**

This Audit included all service users attended ECT suite regularly at Worcestershire Specialist Mental health services over a period of 12 months between April 2022 and April 2023.

Twenty patients were included in this audit for whom data was collected from both electronic and paper records to analyse the percentage of compliance with the Electroconvulsive Therapy Accreditation service (ECTAS) standards with regards to the recommended weekly assessment of mental state via MADRS and the recommended regular assessment of cognition before treatment and every 4 treatment sessions via the MOCA Scale.

**Results:**

The Audit included 20 patients having ECT treatment done regularly over a year.

Overall, 87.32% of the patients were found to have MOCA assessment done before their first ECT session and every 4 treatment sessions as per guidelines. While 96.29% of the patients had MADRAS assessment done weekly or every two treatment sessions as per guidelines.

Regarding MOCA assessment, it has been found that 80% of the patients had MOCA done before their first treatment session. 94.73% of the patients had MOCA done after their 4th treatment session. 89.47% of the patients had MOCA done after their 8th treatment session. And 84.61% of the patients had MOCA done after their 12th treatment session.

With regards to MADRAS, 100% of the patients had MADRAS done before the start of the treatment. 90% of the patients had MADRAS done after the second treatment (1st week). 100% of the patients had MADRAS done after 4th treatment (second week). 100% of the patients had MADRAS done after 6th treatment (third week). 93.33% of the patients had MADRAS done after 8th treatment (4th week). 92.85% of the patients had MADRAS done after the 10th treatment (5th week).

**Conclusion:**

Overall, ECT practice at Worcestershire Specialist Mental health services has been found to be in compliance with the ECTAS guidelines.

The majority of patients had MOCA assessments done regularly every 4 weeks with the highest compliance found to be after the first 4 treatment sessions and the lowest compliance was for the MOCA assessment done before the start of the ECT treatment.

In terms of MADRAS assessment, there was an overall adherence with the guidelines with very few patients missing MADRAS assessment only once over their course of treatment.

